# Bioinspired Sarcomeric Double-Network Hydrogels for Programmable Mechanics with Ultralow Hysteresis

**DOI:** 10.3390/gels12060520

**Published:** 2026-06-10

**Authors:** Yang Luo

**Affiliations:** 1 Department of Mathematics and Physics, North China Electric Power University, Baoding 071003, China; luoyang@pku.org.cn; 2 Hebei Key Laboratory of Physics and Energy Technology, Baoding 071003, China

**Keywords:** double-network hydrogel, ultralow hysteresis, electrical signal, freezeresistance, smart materials

## Abstract

Hysteresis is normally unavoidable in hydrogels under complex external loading conditions due to the intermolecular friction, which usually leads to fatigue. Here, we fabricate a sarcomere-inspired double-network hydrogel made from polyacrylamide, alginate and phytic acid, whose hysteresis can be effectively regulated by preloading. Particularly, due to the synergy of micellization, fibrillation and micro-lubrication, the as-prepared hydrogel displays an ultralow hysteresis (≤0.02%) after it experiences a pre-tensile process at a specific amplitude and strain rate, or even possesses negative hysteresis in the case of low tensile amplitudes or high strain rates. Interestingly, smart responses of the developed hydrogel to cyclic tensile loadingare similar to the mechanical behaviors of sarcomeres in vivo. Likewise, the derived hydrogel with ultralow hysteresis performs reliably even at temperatures as low as −20 °C. The ultralow hysteresis presented by the biomimetic hydrogel with ultralow hysteresis makes it suitable for many engineering fields like electrical sensing with superior reliability (the corresponding electrical signal (ΔR/R_0_) is stable even after 1000 stretching–unstretching cycles). Moreover, the design strategy of hydrogels with programmable hysteresis provides an innovative methodology for the future development of smart high-performance hydrogels.

## 1. Introduction

Highly stretchable, tough and resilient elastomers and gels hold great importance in the fields of flexible electronics, wearable devices, biomedicine, etc. [1,2,3,4,5]. In practice, apart from high strength, stretchability, and toughness, low hysteresis over repeated loading–unloading cycles is critical for soft materials to ensure excellent resilience and resist fatigue [6,7,8,9]. Owing to the poor mechanical robustness of single-network hydrogels—characterized by low strength, limited extensibility, and low fracture energy—researchers have engineered double-network (DN) hydrogels [10] that simultaneously achieve significantly improved toughness and stretchability [11,12,13,14]. Although the interpenetrating network (IPN) structure effectively suppresses crack propagation under mechanical loading, internal intermolecular friction leads to substantial energy dissipation, resulting in pronounced stress–strain hysteresis [15,16]. The material exhibits fatigue behavior, as evidenced by a progressive decline in its mechanical properties under cyclic loading [17,18]. These phenomena primarily arise from intermolecular friction and irreversible rupture of internal bonds in hydrogels subjected to cyclic loading [19,20,21]. Recently, great efforts have been made to reach low hysteresis (<5%) upon cyclic tensile tests via supramolecular microstructures, including multifunctional nano-crosslinkers, coiled protein/polypeptide and polypeptide/metal coordination [22,23,24]. In these cases, fast, efficient, and reversible disassembly/reassembly of supramolecular domains can significantly stabilize the internal microstructure and provide ‘hidden length’ to polymer networks under external stress—a feature that is highly beneficial for reducing intermolecular friction and hysteresis [25,26]. Moreover, the synergistic contribution of abundant noncovalent interactions and topological “hidden length” endows the hydrogel with remarkable stretchability and outstanding toughness [27].

However, “low hysteresis” is not an absolute intrinsic property of a material, but rather acondition-dependent, relative performance metric—strictly defined within specific loading–unloading protocols [28]. On the one hand, it is typically observed under particular mechanical conditions, such as relatively high strain rates and moderate-to-low maximum strains. When the strain rate is reduced or the maximum strain is significantly increased, the hydrogel network gains sufficient time for viscoelastic relaxation and greater conformational reorganization space [29]. Under such conditions, (i) dynamic noncovalent crosslinks—including hydrogen bonds, coordination bonds, and boronate ester bonds—undergo progressive, multi-stage dissociation and reformation [30,31]; and (ii) polymer chains are prone to irreversible slippage or topological rearrangement (e.g., entanglement redistribution), thereby amplifying energy dissipation [32,33]. Consequently, the hysteresis ratio increases and residual strain accumulates—causing the material to deviate from the “low-hysteresis” regime [34,35]. On the other hand, the reported low hysteresis of hydrogelsisnormally recorded under mild conditions (i.e., ambient temperature). The antifreezing or environmentally tolerant properties of hydrogels typically arise from the disruption of hydrogen bonding between water molecules—achieved [36] viahigh concentrations of hydrated ions, organic solvents, orhydrogen-bond-donating functional groups [37,38]. However, these substances can introduce additional sacrificial bonds into the hydrogel network, thereby increasing intermolecular friction and hysteresis under cyclic loading [39]. Achieving low mechanical hysteresis in hydrogels under extreme environmental conditions remains a significant challenge.

The sarcomere—the fundamental contractile unit of striated muscle—exhibits exceptional adaptive mechano-responsiveness to complex, dynamic mechanical loading [40]. Its hierarchical architecture, featuring aligned myofilaments and viscoelastic inter-filamentary matrix, enables sustained high strength, toughness, and fatigue resistance [41] by minimizing intermolecular friction and preserving elastic recoil over repeated cycles [42,43,44,45]. Inspired by this, we developed a biomimetic double-network hydrogel (P-O-ALG-PA) comprising poly(acrylamide-co-octadecyl acrylate), sodium alginate, and phytic acid—integrated via synergistic covalent–ionic–hydrophobic crosslinking [46]. Unlike most reported high-strength and tough hydrogels, the mechanical behavior of P-O-ALG-PA arises from the synergistic interplay between supramolecular aggregates and interfacial lubrication. Remarkably, after a tailored preloading protocol, P-O-ALG-PA exhibits ultralow hysteresis (<0.02%) under cyclic tensile loading—even at low strain rates (~20 mm/min) or large strains up to ~500%. Nowadays, hydrogel-based piezoelectric materials have developed rapidly and possess promising application prospects in the field of implantable bioelectronics [47]. Most existing studies focus on the design of piezoelectric functions and the integration of flexible devices. In this work, a composite hydrogel system is constructed, and its layered fibrous microstructure, low-hysteresis mechanical properties and stable electro-sensing performance are systematically investigated. This study not only enriches the research system of functional hydrogels but also provides new ideas for the development of high-performance flexible sensing hydrogels [28,48]. Moreover, P-O-ALG-PA exhibits autonomous self-healing, exceptional water retention, and robust, substrate-agnostic adhesion to diverse solid surfaces [49]. Collectively, this hydrogel achieves unprecedented environmental adaptability and programmable near-zero hysteresis, establishing a paradigm-shifting design strategy for hysteresis-free intelligent soft materials.

## 2. Results and Discussion

**Figure 1:**
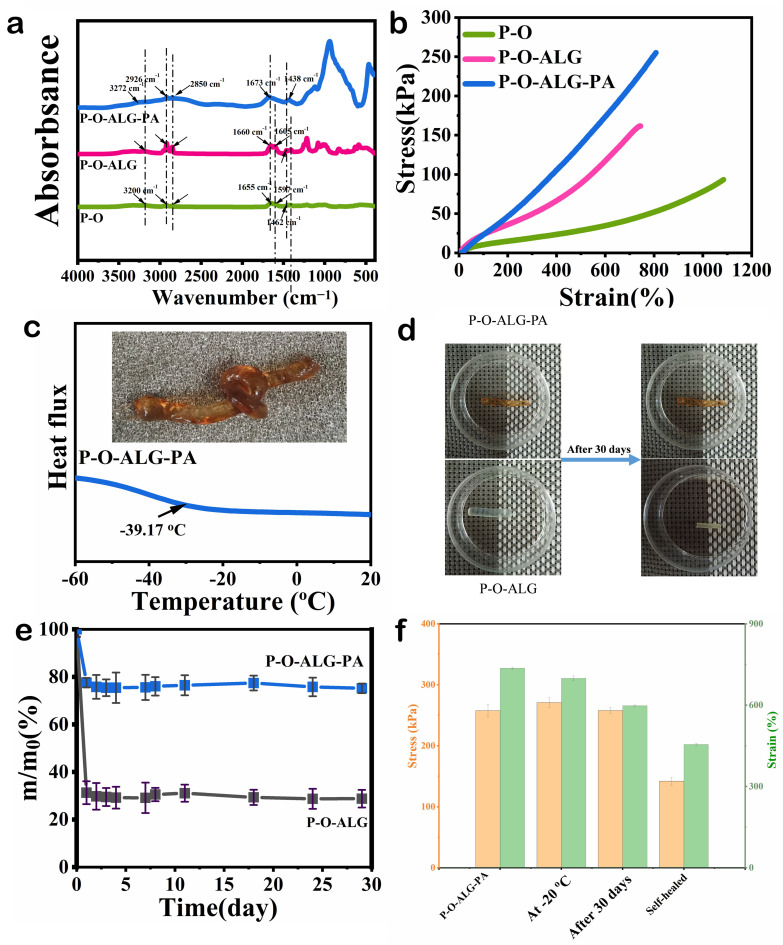
(**a**) FTIR spectra of the P-O-ALG-PA, P-O-ALG, and P-O hydrogels. (**b**) Representative tensile curves of the tested hydrogels. (**c**) DSC curve of the P-O-ALG-PA hydrogel from −60 °C to 20 °C. The inset image shows that the hydrogel remains in a robust gel state at −20 °C and can be freely knotted without fracture. (**d**) Illustration of original P-O-ALG-PA hydrogel, P-O-ALG-PA hydrogel in the air after 30 days, the original P-O-ALG hydrogel, and P-O-ALG hydrogel in the air after 30 days. (**e**) Water-retaining capability and environmental durability of P-O-ALG-PA and P-O-ALG. (**f**) Corresponding stress–strain statistical data for the P-O-ALG-PA hydrogel under the following conditions: as-prepared (room temperature), at −20 °C, after 30 days of ambient air storage at room temperature, and after self-healing following tensile damage.

FTIR spectroscopy was employed to characterize the chemical structures of poly(octadecyl acrylate) (P-O), P-O-alginate (P-O-ALG), and P-O-ALG-phytic acid (P-O-ALG-PA) hydrogels (as Figure 1a shows). All samples exhibit a broad, intense band at 3200–3272 cm^−1^, assigned to O-H stretching vibrations. A characteristic doublet appearing in the range of 2850–2926 cm^−1^ is assigned to the C-H stretching vibrations of -CH_2_- and -CH_3_ groups in the stearyl side chain, confirming the structural integrity of the poly(acrylate-18) main chain in all samples. In the 1438–1673 cm^−1^ region, a broad absorption band—assigned to the asymmetric stretching vibration of carboxylate (-COO^−^) in sodium alginate and overlapped with H-O-H bending of bound water—exhibits progressive blue shifts: from 1590 cm^−1^ in P-O to 1660 cm^−1^ in P-O-ALG and finally to 1673 cm^−1^ in P-O-ALG-PA. This systematic shift reflects enhanced electron withdrawal and strengthened bonding due to coordinative interaction between -COO^−^ groups and phytic acid, corroborating progressive crosslinking and electronic modulation within the ternary network [50]. The band at 1431–1458 cm^−1^ is assigned to the symmetric COO^−^ stretching vibration, coupled with C-H in-plane bending, corroborating structural modulation induced by sodium alginate and phytic acid incorporation [51]. Notably, a strong, broad absorption band emerges exclusively in the P-O-ALG-PA spectrum within 1000–1200 cm^−1^. This feature is assigned to overlapping P-O-C, P-O-H, and PO_4_^3−^ stretching vibrations of phytic acid, together with C-O-C and C-O stretches from alginate’s saccharide rings [52]. Its absence in P-O and P-O-ALG spectra confirms that phytic acid is not merely physically entrapped but is chemically integrated—via covalent bonding and/or coordination—into the hydrogel network, thereby establishing the ternary P-O-ALG-PA architecture.

As shown in Figure 1b, the mechanical strength of the sample increases significantly from P-O to P-O-ALG and further to P-O-ALG-PA, while the fracture strain remains at a relatively high level. This indicates that the incorporation of sodium alginate (ALG) and phytic acid (PA) effectively enhances the material’s mechanical performance. It was important to further explore the low-temperature conditions of P-O-ALG-PA hydrogel. As shown in Figure 1c, the DSC thermogram of the P-O-ALG-PA hydrogel reveals a glass transition temperature of −39.17 °C, significantly below 0 °C, enabling exceptional low-temperature elasticity and structural integrity [53,54]. This exceptionally low glass transition temperature serves as a key indicator of superior antifreezing performance, marking a significant advance in low-temperature-stable soft materials [55]. The outstanding antifreezing capability arises from the synergistic interplay among the three constituent components. Poly (octadecyl acrylate) (POA) acts as an intrinsic plasticizer, depressing the glass transition temperature and maintaining chain mobility at subzero temperatures. Its hydrophobic alkyl chains reduce the unfrozen water content, thereby suppressing ice nucleation. ALG furnishes a robust polysaccharide scaffold that physically impedes ice-crystal propagation and preserves structural integrity. PA forms extensive hydrogen bonds with water molecules, restricting free-water mobility and inhibiting ice growth. Additionally, it serves as a multifunctional crosslinker, reinforcing the alginate network and enhancing mechanical stability under freezing conditions. This hierarchical synergy renders P-O-ALG-PA a promising candidate for low-temperature flexible electronics and implantable biomedical devices. Figure 1d shows that the P-O-ALG-PA hydrogel retains its elasticity after 30 days of storage at 25 °C and 30% relative humidity (RH), whereas the P-O-ALG control undergoes severe dehydration and embrittlement. Figure 1e quantitatively confirms that phytic acid (PA) forms strong hydrogen bonds with water molecules, effectively immobilizing them within the hydrogel network and thereby significantly suppressing water evaporation and loss. Statistical results of the tensile test in Figure 1f revealed that pristine P-O-ALG-PA hydrogel exhibits a high tensile strength of ∼250 kPa and exceptional extensibility (∼650% strain), demonstrating outstanding mechanical robustness and ductility. At −20 °C, the hydrogel retains a tensile stress of ∼270 kPa and strain of ∼600%, demonstrating exceptional low-temperature mechanical stability without embrittlement—consistent with its ultralow glass transition temperature (Tg = −39.17 °C) determined by DSC, confirming the outstanding low-temperature tolerance. After 30 days of storage, tensile stress and strain remain within the same order of magnitude as the pristine hydrogel, confirming exceptional long-term stability. Notably, the hydrogel achieves ~60% recovery of both tensile stress (~150 kPa) and strain (~400%) after autonomous self-healing—without external stimuli—following mechanical rupture. This capability arises from reversible dynamic coordination bonds between phytic acid and oxidized alginate, enabling rapid bond reformation and restoration of structural integrity and mechanical functionality. These results demonstrate that P-O-ALG-PA hydrogel exhibits exceptional cryostability, long-term structural integrity, and autonomous self-healing—making it a promising candidate for flexible electronics, wearable sensors, and biomedical devices operating under harsh conditions.

**Figure 2:**
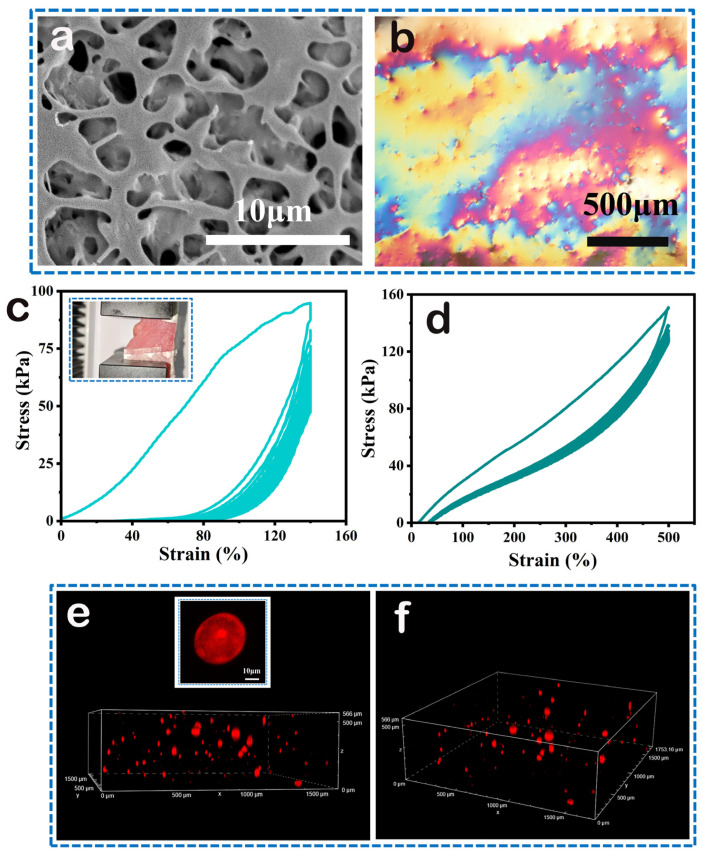
(**a**) Cryo-SEM image of the P-O-ALG-PA hydrogel. (**b**) POM image of the hydrogel. (**c**) Cyclic tensile stress–strain curves of porcine skeletal muscle stretched parallel to the muscle fiber direction, where the inset shows the digital photograph of tested pork tissue. (**d**) Cyclic tensile loading–unloading profiles of the as-prepared hydrogel up to 500% tensile strain. (**e**,**f**) Three-dimensional CLSM images taken from different viewing angles are used to visualize the spatial distribution of fluorescently stained spherical micelles within the hydrogel matrix, where the inset in (**e**) presents a magnified view of an individual stained micelle.

The microscopic porous structure of the synthesized hydrogel was characterized by cryo-scanning electron microscopy (Cryo-SEM). As shown in Figure 2a, the hydrogel forms an interconnected porous network, which provides sufficient free space for the large-scale elongation of polymer chains during stretching. Polarized optical microscopy (POM) was further used to observe the surface anisotropic microstructure of the bulk hydrogel (Figure 1b). Under orthogonal polarized light, the continuous multicolored interference patterns across the entire observation area result from the birefringence effect, directly demonstrating the intrinsic anisotropic characteristics of the prepared hydrogel. Cyclic tensile loading–unloading measurements were conducted to compare the mechanical hysteresis behavior between natural muscle and as-prepared hydrogel. To guarantee data reliability, more than five parallel specimens were measured statistically for both native skeletal muscle tissue and synthesized hydrogel. Figure 2c presents representative cyclic tensile curves of native skeletal muscle tissue as the biological reference sample, while Figure 2d corresponds to the typical cyclic performance of our designed hydrogel. The inset in Figure 2c shows a picture of the muscle tissue undergoing cyclic tensile testing. Notably, the natural muscle fibers and the synthetic hydrogel share an identical characteristic in cyclic mechanics: a conspicuous large hysteresis loop emerges in the first loading–unloading cycle, whereas the subsequent repeated cycles deliver remarkably reduced hysteresis loss with narrow hysteresis loops. Such a consistent evolving law of hysteresis inspired our bioinspired structural design with reference to the sarcomere architecture of natural muscle. Benefiting from the sarcomere-inspired network construction, dynamically reversible dissociation and re-association of noncovalent interactions dominate the energy dissipation pathway. In the first stretching procedure, massive noncovalent crosslinks sequentially rupture to consume external mechanical energy, mimicking the relative sliding of thick and thin myofilaments inside biological sarcomeres and inducing prominent primary hysteresis; during subsequent cyclic stretching, most destructed dynamic bonds can rapidly reconstruct after unloading, resulting in greatly lowered energy dissipation and a minimized hysteresis ratio in succeeding cycles, which reproduces the cyclic mechanical evolution of natural muscle fibers well [3]. Such dynamic bonding evolution matches the reversible contraction–relaxation cycle of sarcomeres well, fundamentally explaining the origin of hydrogel’s muscle-analogous mechanical performance. Three-dimensional laser scanning confocal microscopy (LSCM) was employed to visualize the spatial distribution of functional microdomains within the hydrogel, as depicted in Figure 2e,f. The fluorescentlylabeled micelle spheres are uniformly embedded throughout the entire hydrogel matrix, showing no clear agglomeration. This observation confirms the excellent compatibility and homogeneous dispersion of functional components within the crosslinked polymer network. The inset in Figure 2e clearly reveals individual fluorescentlystained micelle spheres. The uniformly distributed microdomains work in tandem with the oriented anisotropic domains verified by polarized optical microscopy (POM). This synergy facilitates the orderly transfer of stress across the network, suppresses localized stress concentration, and endows the hydrogel with remarkable sarcomere-mimicking properties.

**Figure 3:**
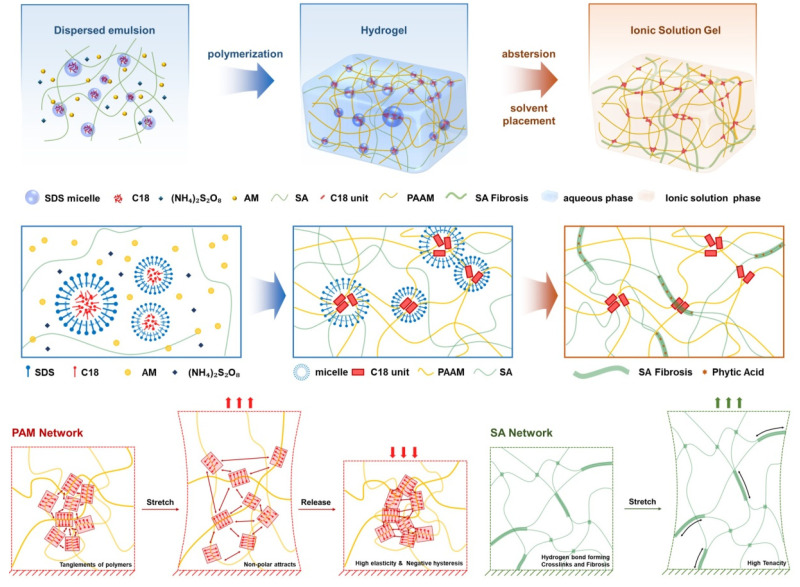
The fabrication procedure of the P-O-ALG-PA hydrogel and representative structural schematics of the hydrogel at key synthesis stages.

Based on experimental evidence, the gelation mechanism is proposed as follows (Figure 3): Free-radical polymerization proceeds without chemical crosslinkers (e.g., MBA). SDS and NaCl synergistically stabilize the oil phase and enable uniform dispersion of OA [56]. Under APS initiation, OA self-assembles into nanoscale micelles—surfactant-stabilized by SDS hydrophilic heads, which serve as dynamic physical crosslinking nodes, anchoring polyacrylamide (PAAm) chains into a robust primary network. Concurrently, sodium alginate constructs a secondary network via hydrogen bonding [6,20]. Subsequent dialysis removes residual SDS, followed by immersion in 70 wt% PA solution, enabling PA infiltration. PA’s phosphate groups form reversible hydrogen bonds with alginate hydroxyls, reinforcing the dual-network architecture and endowing the hydrogel with enhanced mechanical integrity and stimuli-responsive dynamics. PA infiltration introduced abundant, reversible hydrogen bonds between its phosphate groups and hydroxyls of sodium alginate, serving as additional physical crosslinking points [57]. Concurrently, intra- and intermolecular hydrogen bonding among alginate hydroxyls drove hierarchical fibrillar alignment—morphologically and mechanically mimicking sarcomere—thereby significantly enhancing mechanical robustness. The schematic diagram is jointly verified by LSCM, FTIR, and the change in hysteresis rate during cyclic loading–unloading experiments.

**Figure 4:**
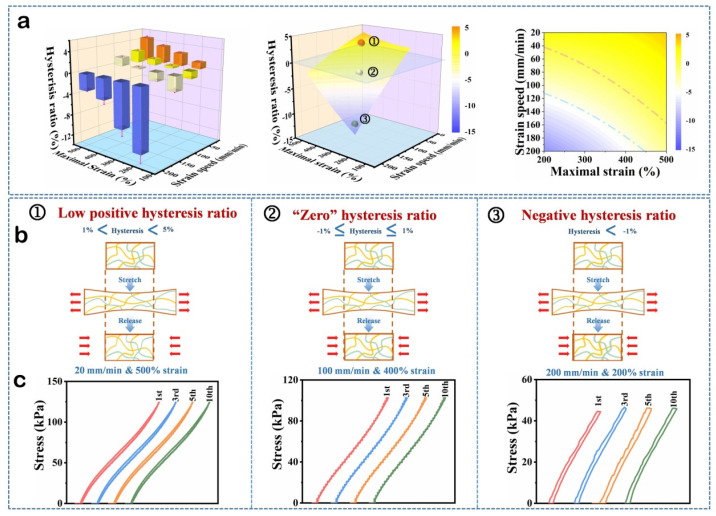
(**a**) Histograms of P-O-ALG-PA at different stretching ratios and strain speeds. Three dimensional fitting surface corresponding with histograms. Corresponding phase image. (**b**) Schematic diagram of loading–unloading test. (**c**) Hysteresis loops of the hydrogel under tensile loading–unloading cycles at crosshead speeds of 20, 100, and 200 mm/min, with corresponding maximum strains of 500%, 400%, and 200%, respectively.

Given its muscle-mimetic anisotropic microstructure, this hydrogel can biomimetically recapitulate the stretch–relaxation cycling behavior of skeletal muscle. Its fatigue behavior and biomimetic mechanical response are quantitatively characterized through the dynamic evolution of hysteresis ratio during cyclic loading–unloading tests. To systematically investigate the viscoelastic hysteresis and biomimetic muscular mechanical response of the P-O-ALG-PA hydrogel, uniaxial cyclic tensile loading–unloading tests were performed using a universal testing machine. A dual-parameter experimental design was employed to probe the coupled effects of maximum strain (200%, 300%, 400%, and 500%) and strain rate (20, 50, 100, and 200 mm/min). Original full stress–strain datasets are supplied in Figure S1. All mechanical parameters are expressed as the mean ± standard deviation, and error bars are marked in Figure 4a. Moreover, multi-batch prepared hydrogel samples present a highly consistent negative hysteresis performance, proving the excellent experimental repeatability and sample-to-sample reproducibility of this unique mechanical phenomenon. We systematically investigated the cyclic loading–unloading behavior of the P-O-ALG-PA double-network hydrogel under combinatorial tensile strains (low/medium/high) and strain rates (high/medium/low). Strikingly, the hysteresis ratio (HR) is not a fixed parameter but exhibits programmable, condition-dependent modulation: HR peaks at <5% under high-strain–low-rate conditions; drops to ~0.2%—near-ideal elasticity—under medium-strain–medium-rate loading; and, most notably, attains a negative value of −8% under low-strain–high-rate conditions, unambiguously indicating net energy return (i.e., unloading work exceeds loading work). Notably, the distinct negative hysteresis behavior achieved at high speed and low strain is originally discovered in this multi-component hydrogel. Up to now, there have been almost no studies reporting negative hysteresis in alginate/phytate-based composite hydrogels. Different from conventional low-hysteresis hydrogels constructed by single dynamic interaction, the triple reversible crosslinking network designed in this work successfully induces the special energy-releasing mechanical property. We propose a biologically inspired “training–testing” framework for the tunable hysteresis behavior, as shown in Figure 4b: high-strain/low-rate loading induces large HR, reflecting structural lag, akin to initial learning resistance; moderate strain/rate yields near-zero HR, signifying optimal stimulus–response synchronization and efficient energy transduction; low-strain/high-rate triggers negative HR, evidencing predictive energy storage/release—reminiscent of anticipatory task execution [58]. Figure 4c clearly demonstrates the hysteresis phenomena under different loading and unloading conditions, effectively validating this rule. Specifically, during the loading–unloading cycles with high strain and low speed, a low-hysteresis characteristic is exhibited; in the cycles with medium speed and medium strain, an ultralow hysteresis rate is shown; and in the cycles with low strain and high speed, a negative hysteresis phenomenon occurs.

**Figure 5:**
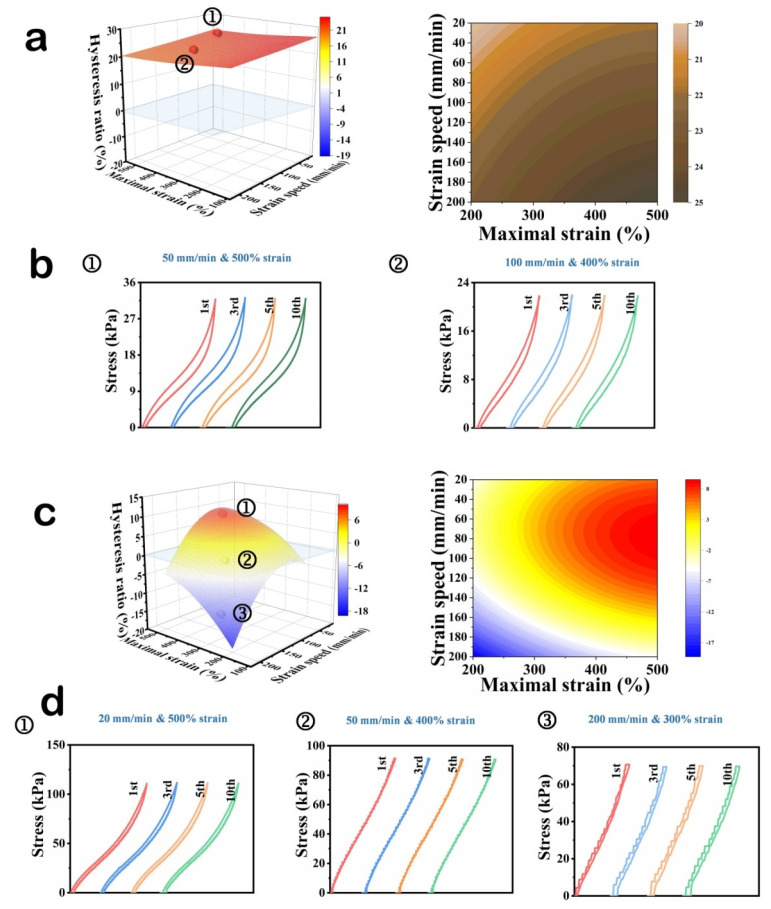
(**a**) Three-dimensional surface graph of P-B-ALG-PA hydrogel. Corresponding phase image. (**b**) Hysteresis loops of the hydrogel under tensile loading–unloading cycles at crosshead speeds of 50 and 100 mm/min, with corresponding maximum strains of 500%, and 400%, respectively. (**c**) Three-dimensional surface graph of P-O-PA hydrogel. Corresponding phase image. (**d**) Hysteresis loops of the hydrogel under tensile loading–unloading cycles at crosshead speeds of 20, 50 and 200 mm/min, with corresponding maximum strains of 500%, 400%, and 300%, respectively.

This HR exhibits a systematic, load-condition-dependent tunability—representing a rare intrinsic programmable mechanical response. To elucidate its structural origin, we fabricated two control hydrogels: P-B-AlG-PA (crosslinked with MBA instead of stearyl acrylate, lacking hydrophobic moieties) and P-O-PA (devoid of alginate but retaining phytic acid and hydrophobic components). Notably, complete omission of OA prevents gelation, confirming its dual role as both covalent crosslinker and hydrophobic microdomain builder. Under identical coupled stretch-ratio/strain-rate cycling protocols, P-B-AlG-PA yields a high, invariant HR (~20% in Figure S2), devoid of rate- or amplitude-dependence (Figure 5a). In Figure 5b, the large enclosed area of the loading–unloading curves for ① and ② verifies the high hysteresis rate of P-B-AlG-PA. This loss of programmability unambiguously identifies the dynamic hydrophobic association network—derived from OA—as the essential structural motif enabling programmable HR regulation.

Cyclic loading–unloading tests of P-O-PA under identical strain-amplitude and strain-rate coupling conditions (Figure S3 and Figure 5c,d) reveal retained rate- and amplitude-dependent hysteresis, yet HR increases markedly (−20% to +10%). This elevation stems directly from the absence of sodium alginate (AlG). AlG synergizes with phytic acid (PA) via directional hydrogen bonding to drive hierarchical nanofibril bundle assembly, establishing a reversible energy-dissipation pathway. In contrast, P-O-PA lacks this structural motif; its sole dissipative network—comprising hydrophobic associations and phosphate–hydrogen bonds between stearyl acrylate and PA—exhibits elevated dissociation energy barriers and sluggish reassociation kinetics, thereby amplifying irreversible interfacial friction and systematically elevating HR. Collectively, these results confirm that the programmable, low-hysteresis mechanical response of P-O-ALG-PA arises exclusively from the precise integration of four components: AM (monomer), OA (dual-function crosslinker/hydrophobe), ALG, and PA, each indispensable to the designed dual-network architecture.

**Figure 6:**
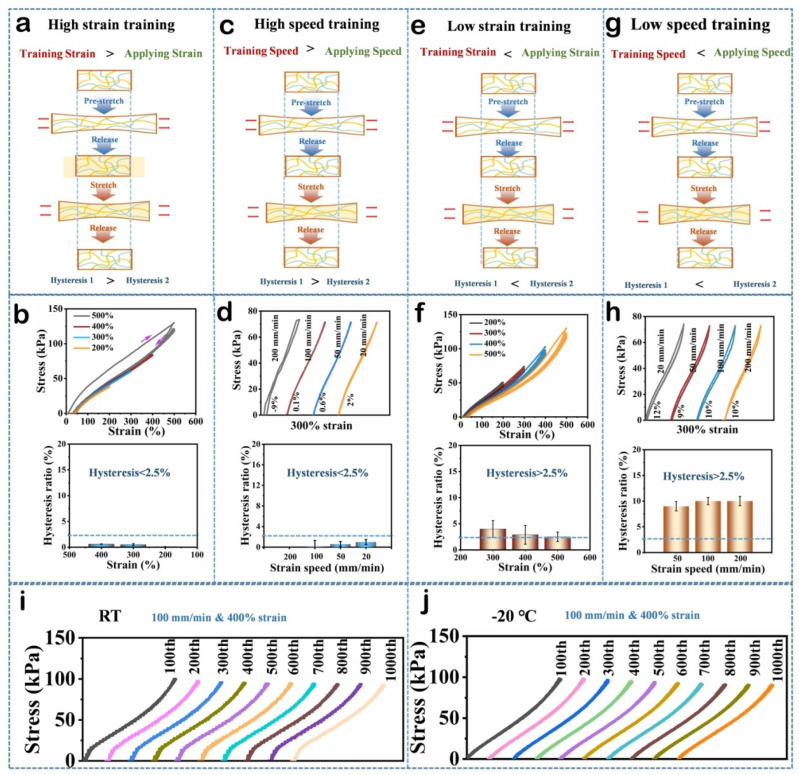
(**a**) Schematic illustration of the “high-magnification learning–low-magnification assessment” paradigm of the P-O-ALG-PA hydrogel. (**b**) Loading–unloading curves at 100 mm/min from the strain of 500% to 200%, and hysteresis ratio responds to each applying strain. (**c**) High-speed training mode of the P-O-ALG-PA hydrogel. (**d**) Loading–unloading curves at a strain of 300% from stretching speed of 200 to 20 mm/min, and hysteresis ratio responds to the applying strain. (**e**) Low-strain training behavior. (**f**) Loading–unloading curves at 100 mm/min from the strain of 200% to 500%, and hysteresis ratio responds to the applying strain. (**g**) Low-strain training behavior. (**h**) Loading–unloading curves at a strain of 300% from stretching speed of 20 to 200 mm/min, and hysteresis ratio responds to the applying strain. (**i**) 1000 cycles of stretching–unstretching curves at room temperature. (**j**) Anti-fatigue measurements at −20 °C.

To systematically validate the programmable “training–testing” response of the hydrogel, we performed coupled experiments: controlled mechanical preconditioning (termed “training”) followed by subsequent mechanical testing (termed “testing”), as schematically illustrated in Figure 6a. A high-intensity training protocol—10 cycles of uniaxial stretching to 500% engineering strain at 100 mm/min—was applied first. Subsequently, stepwise strain-controlled testing was performed at the same crosshead speed (100 mm/min), using progressively decreasing engineering strain levels: 400%, 300%, and 200%. As shown in Figure 6b, the hysteresis ratio (HR) decreased markedly and fell below 2.5%, indicating that intense mechanical preconditioning drives structural reorganization within the dual network, thereby enhancing the reversibility of energy dissipation [59]. Further, we implemented a training–testing protocol in which the training speed exceeded the testing speed to probe the variation inHR, as Figure 6c shows. High-intensity “training” (300% strain, 200 mm/min, 10 cycles) induced HR to −9% (Figure 6d), indicating dynamic bond reconfiguration exceeding instantaneous energy dissipation—i.e., “overfitting.” Subsequent “examination” under milder conditions (200% strain, 100 mm/min) yielded stable HR < 2.5%, demonstrating that overtraining confers broad mechanical adaptability.

Conversely, when the training strain (200%) is lower than the subsequent test strain (from 300% to500%, Figure 6e), the hysteresis ratio (HR) remains elevated (>2.5%) and fails to reach the low values (<2.5%) achieved under matched-strain conditions (Figure 6f). Each stepwise increase in test strain therefore requires one additional preconditioning cycle at that strain level to restore a low HR—revealing that HR is dependent on the learning pattern. The low-rate training mode exhibits the same behavior as shown in Figure 6g,h.

This training–testing paradigm not only demonstrates precise programmability of the hysteresis ratio (HR) through predefined mechanical protocols but also reveals its mechanistic origin: synergistic reorganization of hydrophobic association domains and hydrogen-bonding networks within the dual-crosslinked architecture. Under 400% engineering strain, 100 mm/min crosshead speed, and 25 °C, HR remains stable at 0.20 ± 0.02% over 1000 cycles (Figure 6i), with no measurable degradation in tensile modulus, elongation at break, or elastic recovery. Crucially, this exceptional fatigue resistance is fully retained at −20 °C (Figure 6j), indicating that the hydrogel retains its dynamic activity even at subzero temperatures. Collectively, P-O-AlG-PA hydrogel exhibits mechanically programmable low-hysteresis behavior, establishing a robust material platform for high-fidelity cyclic operation, including real-time soft strain sensing and reversible biomimetic actuation.

**Figure 7:**
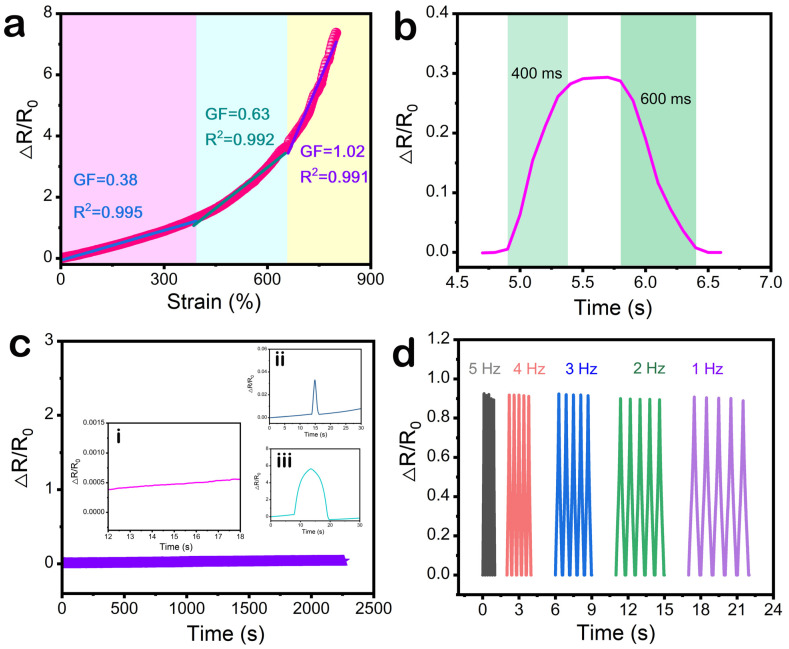
(**a**) The gauge factors of P-O-ALG-PA hydrogel strain sensors in different strain ranges. (**b**) Response and recovery time. (**c**) Baseline drift of hydrogel under static conditions. (i) Magnified view of the baseline signal. (ii) Electrical response signal of the hydrogel under a low strain stimulus of 50%. (iii) Response curve under a large strain stimulus of 700%. (**d**) Frequency-independent sensing behavior under various frequencies (1–5 Hz) at 200% strain.

This low-hysteresis, programmable hydrogel markedly extends the performance frontier of flexible strain sensors. Its tunable hysteresis—rooted in a “training–examination” dynamic bond regulation mechanism—effectively suppresses signal drift and response lag arising from viscous energy dissipation in conventional hydrogels, enabling high-fidelity and highly reproducible strain-to-electrical transduction, which enriches the research scope of hysteresis regulation of functional hydrogels, and also provides a new strategy for preparing high-performance low-delay flexible sensing materials [60]. The electromechanical sensing performance of the hydrogel-based strain sensor was systematically evaluated, as presented in Figure 7. The relative resistance change (ΔR/R_0_) in Figure 7a was depicted as a function of applied tensile strain, revealing a multi-stage linear piezoresistive response across an ultra-wide strain range of 0–800%. Three distinct linear regions with high coefficients of determination (R^2^ > 0.991) were identified, corresponding to gauge factors (GFs) of 0.38 (0–400% strain), 0.63 (400–700% strain), and 1.02 (700–800% strain). This segmented linearity ensures predictable and reliable sensing behavior from subtle deformations to extreme stretching, addressing the common limitation of linearity loss in conventional hydrogel sensors at large strains. The dynamic response of the hydrogel to fast human motion was assessed by monitoring elbow flexion–extension at a 90° angle (Figure 7b). The sensor exhibited a rapid response time of 400 ms and a recovery time of 600 ms, indicating its capability to capture fast joint movements in real time without significant signal lag or hysteresis. Long-term baseline stability is a critical requirement for wearable continuous monitoring applications. As shown in Figure 7c, the sensor maintained an exceptionally stable baseline over 2200 s of continuous testing, with a low drift rate of 0.0013% and a standard deviation of only 0.03%. The magnified baseline in inset (i) further confirms the low noise level of the sensor. To evaluate its detection capability across different strain magnitudes, the sensing signals at 0.5× and 7× strain are presented in insets (ii) and (iii), respectively. The signal-to-noise ratio (SNR) [14] reached 40 dB at 0.5× strain and 86.02 dB at 7× strain, demonstrating a high signal quality even under low deformation conditions. Benefiting from the low noise floor and high sensitivity, the sensor achieved an ultralow limit of detection (LOD) of 0.0237% [9]. The frequency-independent sensing performance of the hydrogel sensor was verified under cyclic stretching at frequencies ranging from 1 Hz to 5 Hz (Figure 7d). The sensor exhibited highly consistent ΔR/R_0_ responses across all tested frequencies. This behavior confirms that the rate of conductive network reconstruction in the hydrogel is well-matched to dynamic deformation rates, supporting stable operation for both low-frequency physiological monitoring (e.g., respiration) and high-frequency limb motion tracking (e.g., rapid joint movements). The hydrogel follows the typical ion-conduction mechanism. Upon the application of an external electric field, the mobile cations migrate through the continuous hydrophilic channels of the swollen polyacrylamide (PAM)/sodium alginate (SA) double-network structure. This migration process results in the formation of conductive pathways, thereby endowing the hydrogel with ionic conductivity rather than electronic conduction [17,22,26].

**Figure 8:**
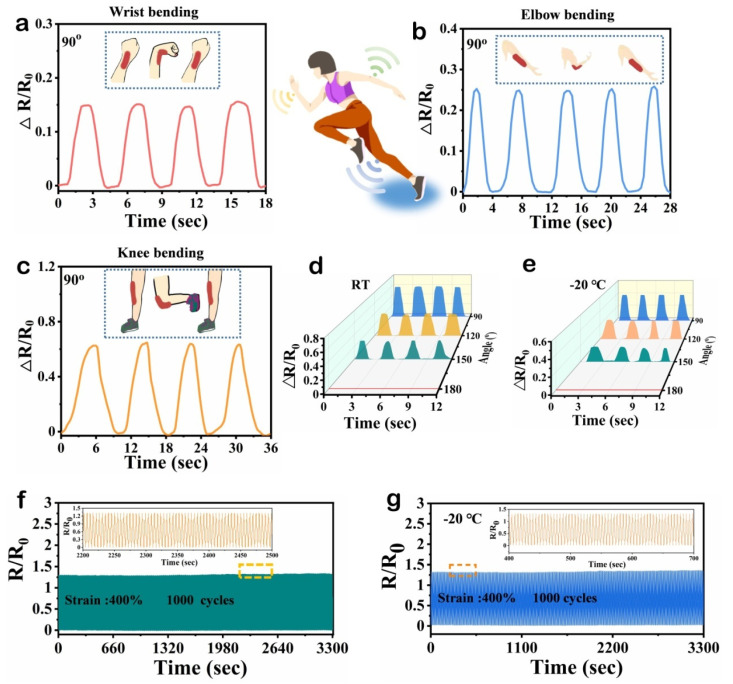
Strain-dependent electrical response of the P-O-SA-PA hydrogel sensor mounted on (**a**) the wrist, (**b**) the elbow, and (**c**) the knee, each bent to 90°. Robust wide-temperature responsiveness is further confirmed by knee flexion across angles at both (**d**) 25 °C and (**e**) −20 °C. Long-term (3.3 × 10^3^ s) electromechanical response of the P-O-SA-PA hydrogel was recorded under cyclic loading–unloading protocols using a universal testing machine at both (**f**) room temperature and (**g**) −20 °C.

In human joint motion tests (wrist, elbow, and knee bent synchronously to 90°) in Figure 8a–c, site-specific signal amplitudes are clearly resolved, with negligible phase lag and no amplitude decay. Robust wide-temperature responsiveness is further confirmed by finger flexion across angles at both 25 °C and −20 °C (Figure 8d,e). Under identical external conditions, the repeated electrical signal responses during knee bending-recovery cycles (Figure S4) and the cyclic experiments of elbow bending at different angles (Figure S5) demonstrate that the hydrogel-based electrical sensor exhibits excellent repeatability [33]. Under rigorous cyclic loading (400% strain, 100 mm/min, 1000 cycles) such as Figure 8f shows, the hydrogel maintains a constant hysteresis of 0.2% and stable, non-drifting conductivity, a direct consequence of synergistic dual-network rearrangement kinetics. This behavior is also retained under long-term cyclic loading at −20 °C (Figure 8g). These attributes confer exceptional large-strain adaptability, cryo-stability, and operational longevity [36]. This stems from the “learning–examination” system-enabled hysteresis tunability, ensuring faithful transduction of dynamic motion trajectories with robust large-strain adaptability, cryo-stability (−20 °C), and fatigue-resistant signal fidelity—enabling high-precision human motion monitoring, extreme-environment flexible electronics, and closed-loop biofeedback systems.

## 3. Conclusions

In summary, we report a chemically unmodified, physically crosslinked hydrogel comprising PAM, OA, ALG, and PA. This material exhibits exceptional antifreezing capability (down to −20 °C), autonomous self-healing, ultralow and programmable hysteresis (hysteresis rate fixed at 0.2% after 1000 cycles), and stable conductivity. It delivers high-fidelity, non-hysteretic, drift-free electromechanical transduction during dynamic joint motions (wrist, elbow, knee, fingers) across a broad temperature range. These integrated properties—originating from synergistic dual-network reconfiguration kinetics and the “training–testing” hysteresis programming strategy—establish it as a robust, mechanism-guided sensing matrix for high-precision wearable motion monitoring and extreme-environment flexible electronics.

## 4. Materials and Methods

### 4.1. Materials

Acrylamide (AM) was used as a monomer to form a primary network copolymerized with oil-soluble monomer octadecyl acrylate (OA). Sodium chloride (NaCl) and sodium dodecyl sulfate (SDS) were used as solubilizing agents to enhance the aqueous dispersibility and surface wetting of C18-functionalized materials. Sodium alginate (ALG) was employed as the secondary network component. Ammonium persulfate (APS) was chosen as the initiator. Phytic acid (PA) 70% (*w*/*w*, aqueous solution) was used as a solution to soak the achieved sample. All reagents in this work were used aspurchased from Aladdin Reagent Inc. without further purification.

### 4.2. Hydrogel Preparation

In order to obtain the ALG hydrogel, the operation steps are as follows: ALG (0.15 g) was dissolved in 5 mL of deionized water under continuous magnetic stirring at room temperature until a homogeneous, viscous solution was obtained. Subsequently, 0.9 g AM was added and uniformly dispersed. Under a water bath temperature of 50 °C, OA, SDS, and NaCl were sequentially added to the aqueous phase at mass fractions of 14%, 35%, and 25% (*w*/*w*), respectively, based on the total mass of solutes [61]. SDS micelles enabled effective solubilization and nanoscale dispersion of OA, establishing a microscopically heterogeneous polymerization medium. APS was then added asathermal initiator, and the mixture was homogenized. The resulting precursor solution was sealed in a polypropylene tube and polymerized thermally at 45 °C to afford the ALG-based hydrogel precursor.

SDS in the SA-based hydrogel impairs both optical clarity and mechanical robustness. To eliminate SDS completely, the hydrogel underwent exhaustive dialysis against deionized water until a constant weight was achieved, followed by solvent exchange—replacing the aqueous phase with a 70 wt% PA solution for 7 h. This protocol yielded a homogeneous, optically transparent, brownish, and flexible P-O-ALG-PA hydrogel.

For comparation, a hydrogel without ALG, ALG hydrogel without soaking in PA, swelled hydrogel, swelled ALG hydrogel, and hydrogel soaked in PA are prepared for future use.

### 4.3. Characterization

Fourier transform infrared spectroscopy (FTIR) was carried out on a Spectrum 100 spectrophotometer (PerkinElmer, Norwalk, CT, USA) with wavenumbers ranging from 4000 to 500 cm^−1^, with a resolution of 1 cm^−1^. The low-temperature phase transition behavior of the hydrogel was studied using differential scanning calorimetry (DSC) with a Q2000 differential scanning calorimeter produced by TA Instruments (New Castle, DE, USA). The sample was placed in a hermetically sealed aluminum crucible. Under a nitrogen flow rate of 20 mL/min, the sample was cooled from 20 °C to −60 °C at a rate of 5 °C/min, and the phase transition temperature and heat flow were recorded. The microstructure was observed by cryo-scanning electron microscopy (Cryo-SEM) (Hitachi-Regulus 8100, Tokyo, Japan) and gold sputtering under an accelerating voltage of 5 kV. Polarized optical microscopy (POM) analysis was performed on a HORIBA-UVISEL Plus (Kyoto, Japan) equipped with orthogonal polarizers at ambient temperature to confirm sample anisotropy. Three-dimensional laser scanning confocal microscopy (LSCM) images were acquired using ZEISS-LSM 880 (Oberkochen, Germany) with the corresponding excitation wavelength for fluorescent labeling at room temperature.

### 4.4. Tensile Testing

A universal tensile testing machine (STS10K model, Xiamen East Instrument Co., Ltd. Xiamen, China)was used to evaluate the mechanical properties of the as-prepared samples. Tests were conducted with a maximum applied load of 20 N. A constant crosshead speed of 100 mm/min was employed for all tensile tests. At least five replicate specimens were tested to ensure statistical reliability, and control samples were tested in parallel under identical conditions. All hydrogel samples were fabricated into cylindrical specimens with a diameter of 5 mm and an initial gauge length of 10 mm. The distance between two clamps of the universal tensile testing machine was set equal to the initial gauge length of samples during testing.

### 4.5. Hysteresis Testing

The hysteresis properties of the samples were measured on the same machine. At a constant strain rate of 100 mm/min, we systematically investigated the path-dependent hysteresis (500% → 100% vs. 100% → 500%) of the hydrogel. Concurrently, at a fixed tensile strain of 300%, we quantified the strain-rate dependence of hysteresis over the range of 20–200 mm/min. This dual-parameter-dependent hysteresis constitutes a programmable, learning-test-mimetic mechanical system.

H_r_ = (W_loading_ − W_unloading_)/W_loading_

where W_loading_ and W_unloading_ are the integral areas of loading and unloading stress–strain curves, respectively. All cyclic tensile measurements were performed on at least five independent identical samples to ensure data reliability.

### 4.6. Electrical Test

The deformation of P-O-ALG-PA hydrogel corresponds to the change in the resistance of the hydrogel, and the function relationship can be established to realize strain sensing. The electrical characterization of the observed sample can be confirmed by a CHI 760E electrochemical workstation with a two-electrode setup. Nickel foam was adopted as current collectors attached to both sides of hydrogel samples, and measurements were implemented at 25 °C with RH ≈ 30%. The conductivity is calculated by the following formula:

σ = L/(R × A)

L (in m), A (in m^2^), and R (in Ω) denote the distance between adjacent electrodes, the cross-sectional area of the hydrogel, and its electrical resistance, respectively.

The hydrogel can be attached to the wrist, elbow or knee of the volunteer and act as a sensor thatconnects with a CHI 760E electrochemical workstation. With the aid of a tensile testing machine, long cycles of testing can be carried out. The electric signal can be recorded following the cycled stretching by the tensile testing machine.

The following formula was used to calculate the variation in resistance:

ΔR/R_0_ = (R − R_0_)/R_0_ × 100%

where R_0_ and R represent the resistance of the original hydrogel and stretching hydrogel, respectively.

The gauge factor, defined as the relative change in resistance per unit strain, is calculated as

GF = (ΔR/R_0_)/ε

where ΔR/R_0_ is the relative resistance change and ε is the applied strain.

The signal-to-noise ratio (SNR) was calculated as

SNR = 20 log_10_(A_signal_/A_noise_)

where A_signal_ is the amplitude of the sensing signal under a small strain, and A_noise_ is the standard deviation of the baseline noise.

Here, SD is the standard deviation of the baseline noise (from Figure 7c), and k is the slope of the calibration curve (GF).

## Data Availability

The original contributions presented in this study are included in the article/Supplementary Materials. Further inquiries can be directed to the corresponding author.

## Supplementary Materials

The following supporting information can be downloaded at https://www.mdpi.com/article/10.3390/gels12060520/s1, Figure S1. Coupling experiment of the P-O-SA-PA hydrogel. Figure S2. Coupling experiment of the P-B-SA-PA hydrogel. Figure S3. Coupling experiment of P-O-PA hydrogel. Figure S4. Repetitive sensing performance of the sensor for knee bending movements. Figure S5. Reproducibility of the sensor during sequential elbow bending: 150–120–90–120–150°. Table S1. Compositions of all the hydrogels in this work. Table S2. The hysteresis ratio with corresponding cycle number under room temperature. Table S3. The hysteresis ratio with corresponding cycle number under −20 °C.
