# Supplementary material for: Bioinspired Sarcomeric Double-Network Hydrogels for Programmable Mechanics with Ultralow Hysteresis
Source: Gels. 2026 Jun 10;12(6):520. doi: 10.3390/gels12060520 (PMC13298709; doi:10.3390/gels12060520)
Supplement: Supplementary file 1 [file gels-12-00520-s001.zip › gels-4339464-supplementary.pdf]

Bioinspired Sarcomeric Double-Network Hydrogels for Programmable Mechanics with Ultralow Hysteresis

Yang Luo [1,2]

[1] Department of Mathematics and Physics, North China Electric Power University, Baoding 071003, China; luoyang@pku.org.cn
[2] Hebei Key Laboratory of Physics and Energy Technology, Baoding 071003, China

Tables:

Table S1. Compositions of all the hydrogels in this work.

Hydrogel	Water (g)	AM (g)	C18 (g)	BIS (mg)	alginic acid (g)	APS (g)	phytic acid (70% solution)
**P-O**	**5**	**0.9**	**0.14**	**0**	**0**	**0.03**	
**P-O-PA**	**5**	**0.9**	**0.14**	**0**	**0**	**0.03**	√
**P-O-SA**	**5**	**0.9**	**0.14**	**0**	**0.15**	**0.03**	
**P-O-SA-PA**	**5**	**0.9**	**0.14**	**0**	**0.15**	**0.03**	√
**P-B-SA-PA**	**5**	**0.9**	**0**	**1**	**0.15**	**0.03**	√

Table S2. The hysteresis ratio with corresponding cycle number under room temperature.

Cycle number	100	200	300	400	500	600	700	800	900	1000
**Hysteresis ratio (%)**	**0.6±0.2**	**0.6±0.05**	**0.3±0.02**	**0.3±0.1**	**0.2±0.1**	**0.2±0.1**	**0.1±0.06**	**0.2±0.07**	**0.08±0.03**	**0.02±0.04**

Table. S3 The hysteresis ratio with corresponding cycle number under -20 ℃.

Cycle number	100	200	300	400	500	600	700	800	900	1000
Hysteresis ratio (%)	0.9±0.5	0.86±0.2	0.8±0.02	0.7±0.1	0.65±0.2	0.6±0.09	0.5±0.06	0.35±0.07	0.2±0.03	0.08±0.05

Figures:

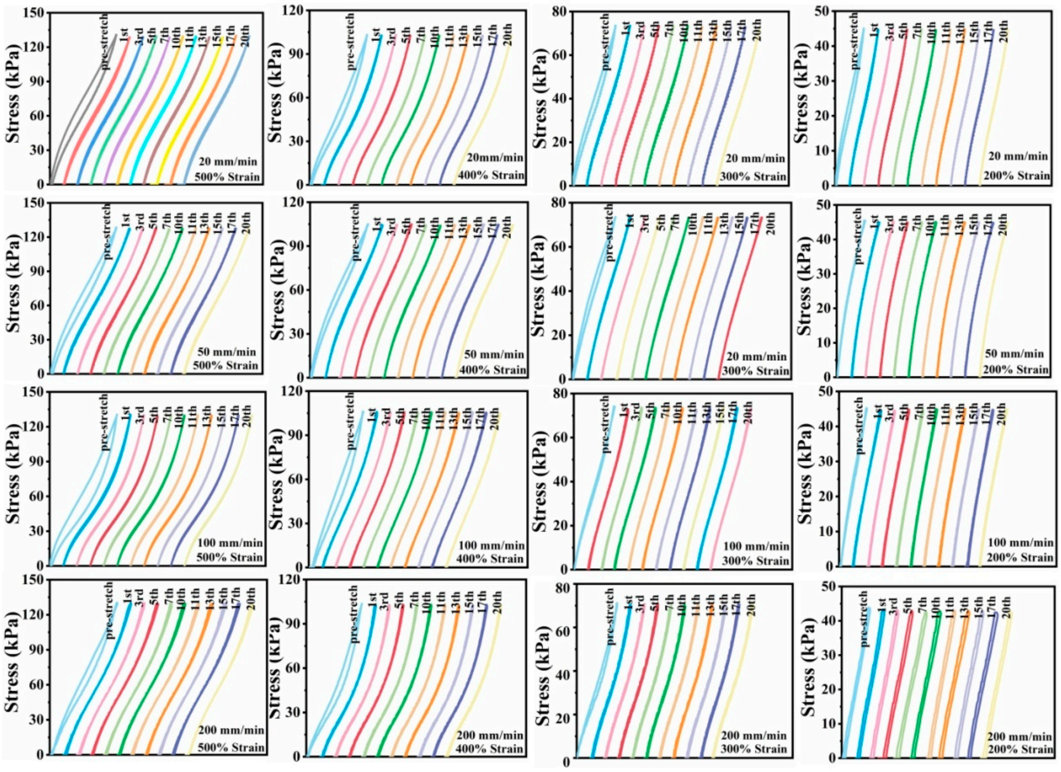

Figure S1. Coupling experiment of the P-O-SA-PA hydrogel.

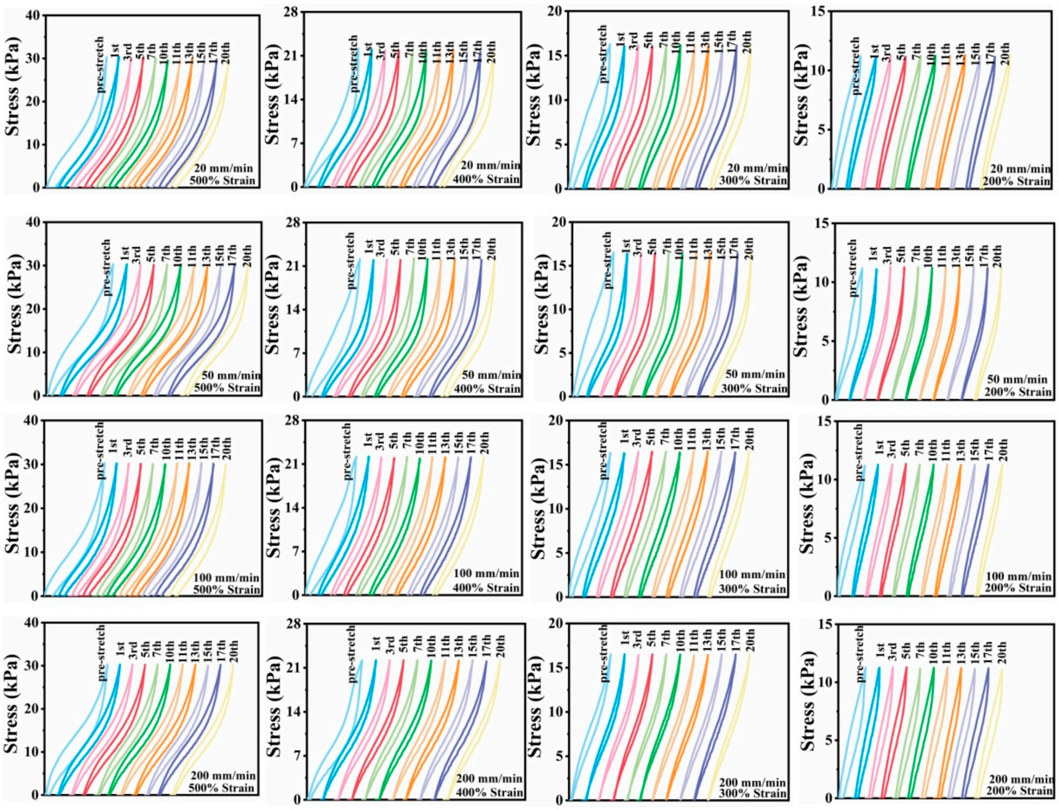

Figure S2. Coupling experiment of the P-B-SA-PA hydrogel.

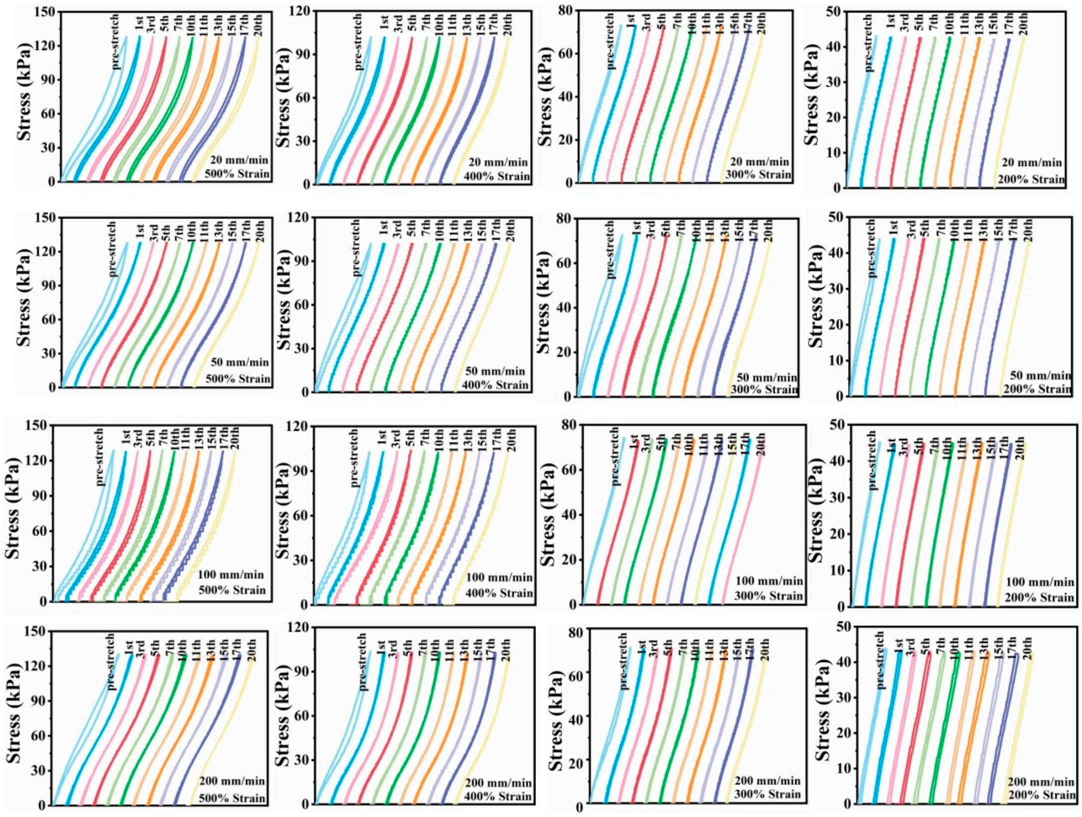

Figure S3. Coupling experiment of P-O-PA hydrogel.

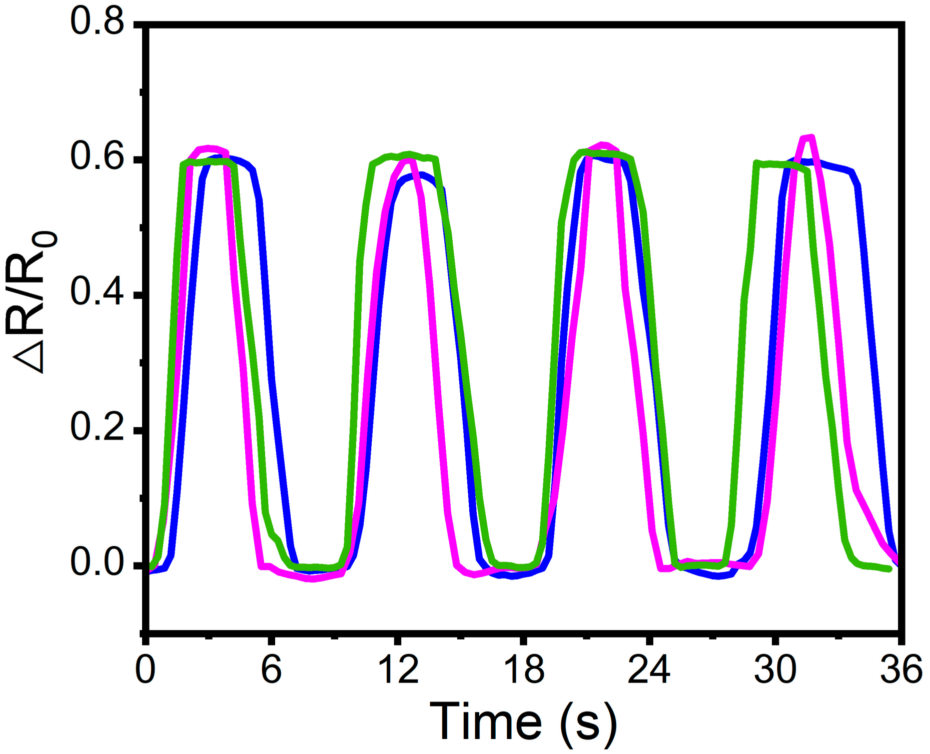

Figure S4. Repetitive sensing performance of the sensor for knee bending movements.

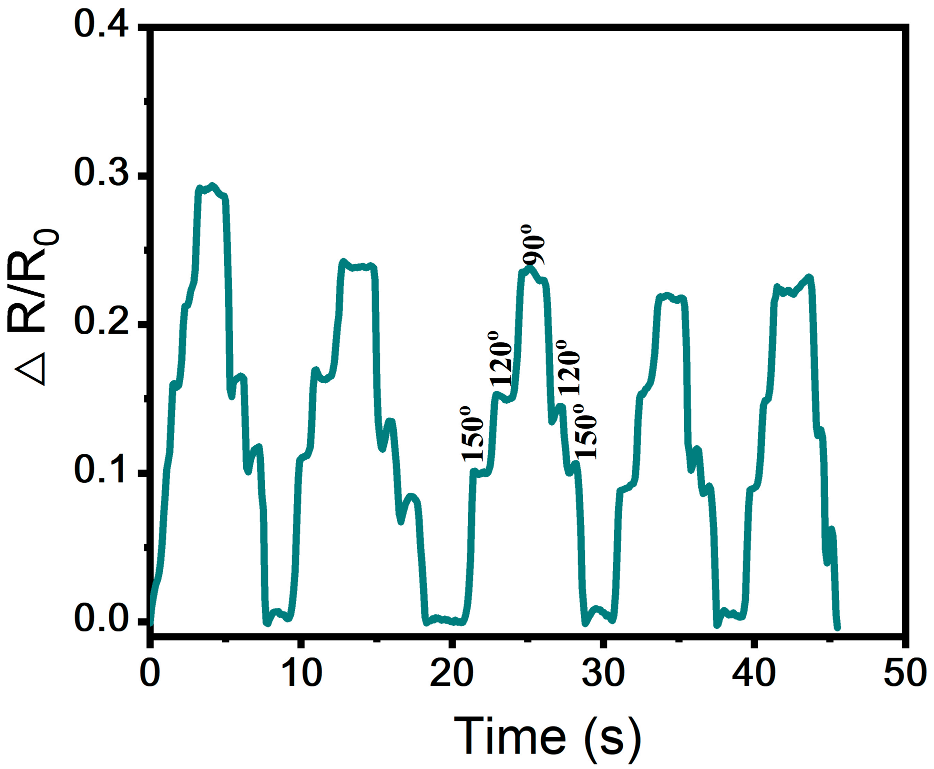

Figure S5. Reproducibility of the sensor during sequential elbow bending: 150° – 120° – 90° – 120° – 150°.
